# Comparative Analysis of Heart Rate Variability and Arterial Stiffness in Elite Male Athletes after COVID-19

**DOI:** 10.3390/jcm13195990

**Published:** 2024-10-08

**Authors:** Mohamed M. Ammar, Noureddine M. Ben Said, Younes N. Ben Said, Ahmed M. Abdelsalam, Sergey P. Levushkin, Aleksey Laptev, Mokhtar Inoubli, Mehdi Chlif

**Affiliations:** 1 Exercise Physiology Department, College of Sport Science and Physical Activities, King Saud University, Riyadh 11362, Saudi Arabia; 2 Biomechanics and Motor Behavior Department, College of Sport Science and Physical Activities, King Saud University, Riyadh 12371, Saudi Arabia; nbensaeed@ksu.edu.sa (N.M.B.S.); amohmed@ksu.edu.sa (A.M.A.); 3 Independent Researcher, Riyadh 11362, Saudi Arabia; 4 Research Institute of Sports and Sports Medicine, Russian University of Sports «GTSOLIFK», Moscow 105122, Russia; levushkinsp@mail.ru; 5 Laboratory of Scientific and Methodological Support for Athletes of National Teams, Institute of Sports and Sports Medicine, Moscow 105122, Russia; laptaleksej@yandex.ru; 6 Research Laboratory of Exercise Performance, Health, and Society, Institute of Sport and Physical Education, Manouba University, La Manouba 2010, Tunisia; moktarmanai@yahoo.fr; 7 EA 3300, Exercise Physiology and Rehabilitation Laboratory, Sport Sciences Department, Picardie Jules Verne University, F-80025 Amiens, France; 8 National Center of Medicine and Science in Sports (NCMSS), Tunisian Research Laboratory Sports Performance Optimization, El Menzah, Tunis 263, Tunisia

**Keywords:** COVID-19, heart rate variability, arterial stiffness, elite athletes, cardiovascular health

## Abstract

This study investigated the long-term cardiovascular effects of coronavirus disease (COVID-19) in elite male athletes by comparing the heart rate variability (HRV), arterial stiffness, and other cardiovascular parameters between those with and without prior COVID-19 infection. **Methods:** This cross-sectional study evaluated 120 elite male athletes (60 post COVID-19, 60 controls) using anthropometric measurements, body composition analysis, pulmonary function tests, HRV analysis, arterial stiffness assessments, hemodynamic monitoring, and microcirculatory function tests. **Results:** Athletes post COVID-19 showed significantly higher lean mass (*p* = 0.007), forced vital capacity (*p* = 0.001), and forced expiratory volume in 1 s (*p* = 0.007) than controls. HRV parameters did not significantly differ between the groups. Post-COVID-19 athletes exhibited peripheral vascular resistance (*p* = 0.048) and reflection index (*p* = 0.038). No significant differences were observed in the blood pressure, cardiac output, oxygen saturation, or microcirculatory oxygen absorption. **Conclusions:** Elite male athletes showed notable cardiovascular resilience after COVID-19, with only minor differences in vascular function. The maintained cardiac autonomic function and improved lung parameters in post-COVID-19 athletes suggests an adaptive response. These findings support the cardiovascular health of elite athletes following COVID-19 but emphasize the importance of continued monitoring.

## 1. Introduction

The COVID-19 pandemic, caused by the SARS-CoV-2 virus, has emerged as a global health crisis with profound implications for various organ systems, particularly the cardiovascular system [1]. Although respiratory symptoms are often the primary clinical presentation, evidence increasingly highlights significant cardiovascular complications, particularly among elite athletes known for their superior cardiovascular fitness due to rigorous training regimens [2,3]. The cardiovascular sequelae of COVID-19 include direct viral invasion of cardiac tissues, systemic inflammatory responses, and dysregulation of the immune system [4]. These pathological mechanisms can result in myocarditis, arrhythmias, endothelial dysfunction, and thromboembolic events, thus contributing to elevated morbidity and mortality rates [5,6]. Given the prevalence of mild or asymptomatic COVID-19, a comprehensive understanding of its cardiovascular impact is essential for its effective management and improved patient outcomes. Elite athletes form a unique group owing to their high physical and cardiovascular demands. Given that individuals resume training and competition after COVID-19, understanding possible changes in their cardiovascular health is vital. This study examined two key indicators: heart rate variability (HRV) and arterial stiffness.

Heart Rate Variability (HRV) is crucial for assessing and enhancing athletic performance because it reflects regulation by the autonomic nervous system. HRV measurement of the intervals between consecutive heartbeats provides insights into the balance between sympathetic and parasympathetic activities. This balance is essential for understanding the physiological state of an athlete and their adaptability to stress [7]. HRV is emerging as a key marker of cardiovascular health in COVID-19, reflecting autonomic function through inter-beat interval variations [8]. COVID-19 can significantly reduce HRV, resulting in increased sympathetic and decreased parasympathetic activity and signaling cardiovascular stress [9,10]. For instance, studies on elite athletes who recovered from COVID-19 showed lower HRV indices, such as the root mean square of successive differences (RMSSD), and elevated heart rates, suggesting altered autonomic regulation [11,12]. Differences in HRV parameters between healthy individuals and COVID-19 patients further underscore HRV’s potential of HRV as a biomarker of cardiovascular compromise [13,14,15].

Arterial stiffness is a crucial marker of vascular health and athletic performance and significantly affects cardiovascular efficiency in athletes. Research has indicated that arterial stiffness is inversely related to left ventricular diastolic function in endurance-trained athletes, suggesting that lower arterial stiffness is associated with better cardiac performance [16]. Arterial stiffness measured by pulse wave velocity (PWV) is a critical determinant of cardiovascular risk [17]. PWV is a critical marker for evaluating cardiovascular risk, as demonstrated by Trimarchi et al. [18]. Incorporating PWV assessments enhances our understanding of vascular health in elite athletes post-COVID-19 [18]. Elevated arterial stiffness is correlated with increased systolic blood pressure, greater cardiac workload, and a higher risk of adverse cardiovascular events, including myocardial infarction and stroke [19,20]. The inflammatory environment and endothelial dysfunction associated with COVID-19 exacerbate arterial stiffness, indicating increased cardiovascular risk during and after infection [21,22]. Meta-analyses have shown that COVID-19 patients have significantly higher carotid-femoral PWV than controls, indicating a potential increase in arterial stiffness and related complications [23]. Although a study of collegiate athletes noted only mild autonomic changes after COVID-19, vascular function assessed by flow-mediated dilation of the brachial artery did not show significant impairment [24]. In contrast, young adults exhibit significant differences in PWV and central systolic blood pressure, indicating that even mild COVID-19 can affect arterial stiffness [25], even without prior comorbidities [26]. 

Despite the growing body of literature on the cardiovascular effects of COVID-19, a critical gap still needs to be addressed regarding the comparative impact on athletes with and without prior infection. The unique physiological status of athletes, which is characterized by enhanced cardiovascular health, may lead to distinct responses to COVID-19. HRV and arterial stiffness are important physiological markers that significantly affect athletic performance. Monitoring and optimizing these parameters can enhance training outcomes, improve recovery, and promote cardiovascular health. Therefore, this study investigated HRV and arterial stiffness in athletes with and without a history of COVID-19. We hypothesized that athletes who contracted COVID-19 would demonstrate lower HRV and increased arterial stiffness than their uninfected counterparts. We employed a cross-sectional study design to test this hypothesis using HRV monitoring and PWV measurements.

This study aimed to deepen our understanding of the cardiovascular consequences of COVID-19 in athletes. By elucidating the differences in HRV and arterial stiffness according to COVID-19 infection status, we sought to provide critical insights into the potential long-term effects of COVID-19 on cardiovascular health in this population. These findings can inform targeted monitoring and management strategies for athletes recovering from COVID-19, enhance return-to-play protocols, and optimize long-term health outcomes. Furthermore, the implications of this study extend beyond individual athletes, potentially influencing public health policies and guiding future research on cardiovascular health in the context of COVID-19.

## 2. Materials and Methods

This cross-sectional study evaluated the physiological parameters of healthy young adults with COVID-19. The study adhered to the principles of the Declaration of Helsinki and was approved by the Institutional Ethics Committee of the Research Institute of Sports and Sports Medicine, Russian University of Sports “GTSOLIFK” (Registration code: ECEC RUS 3194/2024). All participants provided informed consent prior to inclusion in the study.

### 2.1. Participants

The study involved 120 elite male athletes aged 18–35 years (M = 22.4, SD = 3.5) who provided written informed consent and were trained for more than 20 metabolic equivalents of task (MET) hours per week [27,28]. Participants were classified into two groups: 60 athletes with a history of COVID-19 (COVID group) and 60 without COVID-19 (control group). The COVID group included athletes who tested positive by PCR and fully recovered at least three months before the study. The control group consisted of athletes without a history of COVID-19, as confirmed by the negative antibody tests. All participants actively engaged in competitive sports at the professional level, ensuring a uniform elite training status. The exclusion criteria for both groups were any history of neurological disorders, chronic health conditions unrelated to COVID-19, pathological arrhythmias, or cardiovascular diseases. The study was conducted on 30 Jun 2021, before the availability of COVID-19 vaccines, and the average time from positive COVID-19 PCR results was 25 ± 11 days. At the time of evaluation, all athletes, including those in the COVID group, were symptom-free and showed no pathological findings on echocardiography, electrocardiography, or blood tests. This design allowed for a focused examination of the potential long-term impact of COVID-19 on the cardiovascular system in elite athletes. 

### 2.2. Procedure

Participants underwent a comprehensive health evaluation in a temperature-controlled room (22–24 °C). They abstained from caffeine and alcohol consumption, exercised vigorously for 24 h before testing, and rested for 10 min upon arrival to stabilize their physiological parameters. The assessment protocol followed a specific sequence: anthropometric measurements (weight, height, and BMI), body composition using bioelectric impedance (Bioelectrical impedance device: Tanita MC 718, Tanita Corporation, Tokyo, Japan), pulmonary function tests (Spirolab III” turbine flowmeter, MIR, Rome, Italy), autonomic regulation assessment by heart rate variability (Polar H10, Polar Electro, Kempele, Finland), arterial stiffness and vascular compliance using pulse wave analysis with photoplethysmographic (PPG) sensors (Finapres Vantage 4100 model, Finapres Medical Systems, Amsterdam, Netherlands), hemodynamic monitoring (PF07, Manatec Biomedical, Paris, France), and microcirculation and pulse oximetry (Quark CPET, Cosmed, Rome, Italy; and Onyx^®^ II 9550, Nonin Medical, Plymouth, United States). The Finapres system was used to capture synchronous biosignals, enhancing the accuracy of our autonomic dysfunction assessment. Recent studies further support the use of Finapres in evaluating COVID-19 patients with COVID-19, illustrating its broad applicability in such analyses [29,30]. All measurements were conducted by trained professionals following standardized protocols. 

### 2.3. Anthropometric Measurements

With participants dressed in light clothing and without shoes, weight was measured to the nearest 0.1 kg using a digital scale (Tanita MC 718, Tanita Corporation, Tokyo, Japan), and height was measured to the nearest 0.1 cm using a stadiometer (Holtain, Crymych, Dyfed, UK). Body mass index (BMI) was calculated by dividing weight in kilograms by height in meters squared (kg/m^2^).

### 2.4. Body Composition Analysis

Body composition was analyzed using bioelectrical impedance analysis (BIA), a noninvasive technique that can provide detailed insights into body composition. This method has been validated against reference techniques [31]. A Tanita body analyzer (Tanita MC 718, Tanita Corporation, Tokyo, Japan) was used for this purpose. The participants stood with their feet shoulder-width apart and their arms slightly extended while the electrodes were placed on their right hands and feet. A small electrical current was used to measure the resistance and determine the body composition. Key metrics include fat-free mass (FFM), fat mass (FM), total body water (TBW), and muscle mass, providing information on metabolic function and cardiovascular risk [32]. The phase angle (PhA) was also been measured as a proxy for cellular health, with higher values indicating better health outcomes [33].

### 2.5. Pulmonary Function

Pulmonary function was assessed using spirometry following the guidelines established by the American Thoracic Society (ATS) and the European Respiratory Society (ERS) [34]. A SpiroLab III spirometer (Spirolab III” turbine flowmeter, MIR, Rome, Italy) was used. The participants performed standardized breathing maneuvers to measure the forced vital capacity (FVC), forced expiratory volume in one second (FEV1), maximum expiratory flow (PEF), and FEV1/FVC ratio. Participants performed at least three measurements to ensure their validity and reproducibility. The spirometer was calibrated before each session to ensure its precision. The predictive values were obtained from a study by Roca et al. [35].

### 2.6. Autonomic Regulation

HRV provides a picture of the dynamic balance between the sympathetic and parasympathetic branches of the autonomic nervous system (ANS). It is an essential index of pathological changes and is used to diagnose cardiovascular risk in humans. 

Heart rate variability (HRV) was evaluated using a Polar H10 heart rate monitor (Polar H10, Polar Electro, Kempele, Finland). The participants rested in the supine position for 10 min before the measurement to establish a relaxed baseline. HRV was recorded for 5 min, and the data were analyzed using Kubios HRV software (Kubios, Kubios Oy, Kuopio, Finland), which is known for its precision. HRV parameters included high-frequency power (HF) (0.15 to 0.40 Hz), low-frequency power (LF) (0.04 to 0.15 Hz), and the LF/HF ratio, reflecting autonomic balance (Task Force of the European Society of Cardiology and the North American Society of Pacing and Electrophysiology, 1996). The standard deviation of the NN intervals (SDNN) was also measured, indicating variability in the heart rate and autonomic function [36].

### 2.7. Arterial Stiffness and Vascular Compliance

Arterial stiffness and vascular compliance were assessed using pulse wave analysis with photoplethysmography (PPG) sensors (Finapres Vantage 4100 model, Finapres Medical Systems, Amsterdam, Netherlands) following established protocols [37]. Participants rested for 10 min in a temperature-controlled room before measurements were taken over 5 min, with the PPG sensor attached to the index finger of the non-dominant hand. The PPG waveform was recorded at 1000 Hz and analyzed using validated software (Finapres Vantage 4100 Data Management Software) [38]. The critical indices analyzed included the tension index; stiffness index; reflection index; augmentation index; and b/a, c/a, d/a, and e/a ratios, providing comprehensive insights into arterial health [38,39,40,41]. This noninvasive approach can provide valuable information for cardiovascular risk stratification in clinical and research settings [42].

### 2.8. Hemodynamic Monitoring

Blood pressure measurements, including systolic blood pressure (SBP) and diastolic blood pressure (DBP), were obtained using a validated automatic oscillometric monitor (PF07, Manatec Biomedical, Paris, France ) following the American Heart Association guidelines [43]. Participants rested quietly in a seated position for five minutes before three consecutive measurements were taken at 1 min intervals. The means of the second and third readings were used for analysis. Additional hemodynamic parameters, including peripheral vascular resistance (PVR), cardiac output (CO), and volume flow index, were assessed using a noninvasive impedance cardiography device (PhysioFlow PF07, Manatec Biomedical, Paris, France). This device has been validated against invasive methods [44] and provides reliable estimates of cardiac function [45]. All measurements were performed in triplicate by trained personnel who were blinded to the study hypotheses. Stringent quality control measures were implemented. Data were analyzed using appropriate statistical methods based on distribution, as assessed by Shapiro–Wilk tests, and reliability was evaluated using intraclass correlation coefficients [46].

### 2.9. Microcirculation and Oxygenation

Using Onyx^®^ II 9550 (Onyx® II 9550, Nonin Medical, Plymouth, United States), pulse oximetry was used to assess oxygenation and provide real-time SpO_2_ monitoring. This device is recognized for its high accuracy, validity, and reproducibility [47]. 

The aerobic capacity index (ACI) measures the efficiency of oxygen uptake and utilization in the microcirculatory system and is expressed in milliliters of oxygen per minute per square meter (mL O_2_/min/m^2^) of the body surface area. This index is vital for assessing the oxygen absorption capacity of the microcirculation system [48] and reflects the overall cardiovascular health and physical fitness [49]. Maximal oxygen consumption (VO_2_ max) was determined using indirect calorimetry during a graded exercise test on a treadmill (Quark CPET, Cosmed, Rome, Italy). 

### 2.10. Ethical Considerations

Ethical approval was obtained from the Institutional Review Board of the Research Institute of Sports and Sports Medicine, Russian University of Sports “GTSOLIFK” (Registration code: ECEC RUS 3194/2024). Participants provided written informed consent and were informed of their right to withdraw from the study without penalty. Data confidentiality was maintained, and the results were anonymized for analysis in accordance with the principles outlined in the Declaration of Helsinki.

### 2.11. Data Analysis

A priori power analysis was performed using G*Power software (version 3.1.9.7) to determine the appropriate sample size [50]. This analysis ensured adequate power to detect statistically significant effects, thus minimizing the risk of type II errors. A total of 82 participants, with 41 in each group, were required to utilize an effect size of Cohen’s d = 0.5, with a power of 1-β and an alpha level (α) of 0.05. The study included 120 participants (60 in each group), ensuring sufficient power to reduce the risk of type II errors and to improve the generalizability of the findings to the target population of athletes. Statistical analyses were performed using IBM SPSS Statistics for Windows, version 27. Descriptive statistics (mean and standard deviation) were calculated for all variables. Independent t-tests were used to compare the means of continuous variables between the COVID-19 and control groups. Before conducting the t-tests, the assumption of normality was assessed using the Shapiro–Wilk test, and the assumption of homogeneity of variance was evaluated using Levene’s test. The significance level was set at *p* < 0.05. The effect sizes were calculated using Cohen’s d [51]. Additionally, 95% confidence intervals were calculated for all comparisons to quantify the precision of the estimates.

## 3. Results

### 3.1. Anthropometric Measurements

The results (Table 1) showed no significant differences in age (t = 1.65, *p* = 0.101, 95% CI [−0.46, 5.26], ES = 0.30), weight (t = 1.45, *p* = 0.150, 95% CI [−1.31, 8.91], ES = 0.34), height (t = 1.55, *p* = 0.124, 95% CI [−0.08, 0.64], ES = 0.63), body mass index (BMI) (t = 0.00, *p* = 1.000, 95% CI [−0.54, 0.54], ES = 0.00), fat mass (t = −0.11, *p* = 0.913, 95% CI [−1.84, 1.64], ES = −0.02), total body water (t = 0.16, *p* = 0.871, 95% CI [−1.18, 1.38], ES = 0.03), muscle mass (t = 0.19, *p* = 0.849, 95% CI [−0.96, 1.16], ES = 0.03), phase angle (t = 0.84, *p* = 0.403, 95% CI [−0.27, 0.67], ES = 0.15), years of training (t = 0.82, *p* = 0.413, 95% CI [−0.21, 0.51], ES = 0.15), and hours of training per week (t = −0.56, *p* = 0.578, 95% CI [−0.46, 0.26], ES = −0.10). A significant difference was found only in lean mass, with individuals with COVID-19 having a higher lean mass than those without (t = 2.41, *p* = 0.017, 95% CI [0.62, 6.38], ES = 0.44).

### 3.2. Pulmonary Function

The results of the pulmonary function test comparing athletes with and without prior COVID-19 infection are presented in Table 2. Significant differences in forced vital capacity (FVC) and forced expiratory volume in one second (FEV1) were observed. Athletes who had COVID-19 showed higher FVC (5.9 ± 0.8 L) compared with those who did not (5.3 ± 1.1 L), with a t-value of 3.40 (*p* = 0.001, 95% CI [0.24, 0.96], Cohen’s d = 0.62). Similarly, FEV1 was higher in athletes who had COVID-19 (5.0 ± 0.7 L) than in those who did not (4.6 ± 1.0 L), with a t-value of 2.75 (*p* = 0.007, 95% CI [0.10, 0.70], Cohen’s d = 0.49).

No significant differences were found for the FEV1/FVC ratio (t = −0.55, *p* = 0.584, 95% CI [−4.80, 2.80], Cohen d = −0.10), peak expiratory flow (PEF) (t = −0.90, *p* = 0.370, 95% CI [−1.14, 0.43], Cohen d = −0.18), FEV1 percentage of predicted FEV1 (t = −1.54, *p* = 0.126, 95% CI [−8.89, 1.12], Cohen d = −0.27), forced expiratory flow at 25–75% (FEF25–75%) (t = −1.38, *p* = 0.171, 95% CI [−0.72, 0.13], Cohen d = −0.27), and maximal voluntary ventilation (MVV) (t = 1.23, *p* = 0.220, 95% CI [−3.02, 13.02], Cohen d = 0.22). These results indicate that prior COVID-19 infection was associated with higher FVC and FEV1 values, whereas other pulmonary function parameters remained unaffected.

### 3.3. Autonomic Regulation

The heart rate variability parameters of athletes with and without prior COVID-19 infection are presented in Table 3. The results did not show significant differences between the two groups. The heart rate was slightly lower in athletes who had COVID-19 (79.3 ± 9.5 bpm) than in those who did not (81.5 ± 12.7 bpm), but the difference was not statistically significant (t = −1.01, *p* = 0.316, 95% CI [−6.51, 2.11], Cohen’s d = −0.20). The high frequency (HF) percentage was higher in the COVID-19 group (32.7 ± 7.1%) than in the non-COVID-19 group (31.4 ± 7.4%), although this difference was not significant (t = 0.94, *p* = 0.349, 95% CI [−1.39, 3.79], Cohen’s d = 0.18). The (LF) was comparable between the two groups (35.7 ± 9.1% for the COVID-19 group vs. 35.1 ± 11.8% for the non-COVID-19 group), with no significant difference (t = 0.29, *p* = 0.770, 95% CI [−3.40, 4.60], Cohen’s d = 0.06). The LF/HF ratio remained identical between the groups (1.2 ± 0.5 for both), showing no difference (t = 0.00, *p* = 1.000, 95% CI [−0.21, 0.21], Cohen’s d = 0.00). Lastly, the standard deviation of NN intervals (SDNN) was slightly lower in the COVID-19 group (51.9 ± 10.5 ms) than in the non-COVID-19 group (53.5 ± 16.1 ms). However, this difference was not significant (t = −0.56, *p* = 0.576, 95% CI [−6.97, 3.77], Cohen’s d = −0.11).

### 3.4. Arterial Stiffness

Arterial stiffness was evaluated using several indices to compare athletes who had COVID-19 with those who did not (Table 4). The tension index did not show significant differences between COVID-19 athletes (M = 128.0, SD = 56.2) and those without COVID-19 (M = 128.9, SD = 80.6), t = −0.07, *p* = 0.945, with an effect size (Cohen’s d) of −0.01, and a 95% confidence interval (CI) of [−29.66, 27.86]. Similarly, the stiffness index did not differ significantly between the groups (COVID-19: M = 6.4, SD = 0.7; no COVID-19: M = 6.1, SD = 0.9), t = 1.86, *p* = 0.065, although the effect size was 0.35, indicating a small effect with a 95% CI of [−0.02, 0.62]. However, the reflection index revealed a significant difference, with athletes who had COVID-19 showing lower values (M = 25.8, SD = 2.7) than those without COVID-19 (M = 27.2, SD = 4.3), t = −2.10, *p* = 0.038, and an effect size of −0.40, suggesting a minor effect with a 95% CI of [−2.73, −0.08]. Finally, there was no significant difference in the augmentation index between the two groups (M = 1.0, SD = 0.1), t = 0.00, *p* = 1.000, with an effect size of 0.00 and a 95% CI of [−0.03, 0.03].

Vascular compliance was assessed using several ratios, and no significant differences were found between athletes with and without COVID-19 (Table 4). The blood flow ratio b/a was identical in both groups (M = −1.1, SD = 0.2), t = 0.00, *p* = 1.000, with an effect size (Cohen’s d) of 0.00 and a 95% CI of [−0.08, 0.08]. The c/a compliance ratio also showed no significant difference (M = −0.1, SD = 0.1), t = 0.00, *p* = 1.000, with an effect size of 0.00 and a 95% CI of [−0.03, 0.03]. Similarly, the dilation ratio d/a (both M = −0.3, SD = 0.0), t = 0.00, *p* = 1.000, with an effect size of 0.00, and a 95% CI of [−0.01, 0.01] and the elasticity ratio e/a (both M = 0.3, COVID-19 SD = 0.1, no COVID-19 SD = 0.2), t = 0.00, *p* = 1.000, with an effect size of 0.00, and a 95% CI of [−0.06, 0.06] showed no significant differences.

### 3.5. Blood Pressure

Hemodynamic parameters were assessed in athletes with and without prior COVID-19 (Table 5). There were no significant differences in the mean blood pressure (MBP), systolic blood pressure (SBP), diastolic blood pressure (DBP), cardiac output (CO), or stroke volume index (SVI) between the groups. The mean blood pressure was 89.8 ± 8.7 mmHg for athletes with COVID-19 and 89.4 ± 7.7 mmHg for those without COVID-19, with a t-value of 0.27 (*p* = 0.785, 95% CI [−1.99, 2.59], Cohen’s d = 0.04). Systolic blood pressure showed similar results (COVID-19:125.0 ± 12.0 mmHg; no COVID-19:124.3 ± 11.9 mmHg), t = 0.29, *p* = 0.772, 95% CI [−3.54, 4.94], Cohen’s d = 0.06. Diastolic blood pressure was also comparable (COVID-19:72.1 ± 8.7 mmHg; no COVID-19:71.9 ± 7.9 mmHg), t = 0.14, *p* = 0.886, 95% CI [−2.37, 2.77], Cohen’s d = 0.02.

Vascular resistance and cardiac output were assessed in athletes with and without a history of COVID-19. For peripheral vascular resistance (PVR), a significant difference was found between athletes who had COVID-19 (M = 1085.4, SD = 123.7) and those who did not (M = 1142.5, SD = 166.2); t = −2.00, *p* = 0.048, with an effect size (Cohen’s d) of −0.37, indicating a small effect, with a 95% confidence interval (CI) of [−113.50, −0.50]. On the contrary, no significant differences in cardiac output were observed between the two groups (COVID-19: M = 6.7, SD = 0.7; no COVID-19: M = 6.4, SD = 0.9), t = 1.89, *p* = 0.061, with an effect size of 0.34, suggesting a small effect and a 95% CI of [−0.01, 0.61]. Furthermore, the stroke volume index was not significantly different between athletes with COVID-19 (M = 3.3, SD = 0.3) and those without it (M = 3.3, SD = 0.4), t = 0.00, *p* = 1.000, with an effect size of 0.00, indicating no effect and a 95% CI of [−0.10, 0.10].

### 3.6. Oxygen Saturation

When assessing SpO_2_ levels and oxygen absorption in the microcirculation system, no significant differences were observed between athletes who had COVID-19 and those who did not (Table 5). For SpO_2_ levels, athletes with COVID-19 had a mean SpO_2_ of 94.4 (SD = 1.6) compared with 94.5 (SD = 1.8) for those without COVID-19; t = −0.31, *p* = 0.756, with an effect size (Cohen’s d) of −0.06, indicating a minimal effect, and a 95% confidence interval (CI) of [−0.85, 0.65]. Similarly, oxygen absorption in the microcirculation system, measured as the aerobic capacity index (ACI), did not show significant differences between the two groups, with a mean of 299.5 (SD = 46.3) for athletes who had COVID-19 and 301.9 (SD = 41.1) for those who did not; t = −0.30, *p* = 0.766, with an effect size of −0.05, also indicating a minimal effect, and a 95% CI of [−15.16, 10.56].

## 4. Discussion

This study comprehensively examined cardiovascular and physiological differences between elite male athletes with and without COVID-19. Our findings offer significant information on the potential effects of COVID-19 in highly trained individuals, particularly on autonomic regulation, arterial stiffness, vascular compliance, hemodynamics, and microcirculation. One of the most striking conclusions of this study is the remarkable cardiovascular resilience of elite athletes following COVID-19, with only subtle differences observed in selected vascular parameters. This resilience suggests that elite athletes’ high levels of physical conditioning may mitigate the adverse cardiovascular effects associated with COVID-19.

### 4.1. Anthropometric Data and Body Composition

Our study identified significant differences in lean mass between athletes who contracted COVID-19 and those who did not, with the COVID-19 group exhibiting greater lean mass (Table 1). These findings indicated that COVID-19 did not substantially affect body composition in elite athletes, which is consistent with the findings of Hull, Wootten [52]. The preservation of lean mass among these athletes suggests that high-level training can protect against muscle wasting, which is commonly associated with severe cases of COVID-19 in the general population [53]. The ability to maintain body composition contributes to the relatively rapid return to high-level performance observed in many athletes’ post-infection. To support our findings, studies of NCAA Division I collegiate football players found no significant differences in body composition before and after COVID-19 restrictions, indicating that such restrictions did not significantly affect these athletes [54].

Furthermore, research on patients post COVID-19, including athletes, has shown that lean body mass remains high and is not significantly correlated with hospitalization history, illness duration, or level of physical activity [55]. A global study on training practices during lockdowns noted that many athletes maintained or increased their training intensity, potentially explaining why COVID-19 athletes maintained or increased lean mass [56]. These studies highlight the complex interaction among COVID-19, lifestyle changes, and body composition, underscoring the need for further research to understand the underlying mechanisms and long-term effects.

### 4.2. Spirometric Data

Spirometric data analysis revealed notable differences in pulmonary function parameters between the COVID-19 and control groups (Table 2). Specifically, athletes with a history of COVID-19 exhibited significantly greater forced vital capacity (FVC) (*p* = 0.001) and forced expiratory volume in one second (FEV1) (*p* = 0.007). These findings suggest that although COVID-19 may have acute effects on respiratory function, elite athletes can maintain a higher level of pulmonary capacity than their non-infected counterparts, possibly because of their superior baseline fitness and resilience. However, it is essential to note that the FEV1/FVC ratio did not differ significantly between the groups (*p* = 0.584), indicating that the overall airflow limitation was not compromised. These findings align with those of Mohr and al [57], who reported that although athletes exhibited some reductions in spirometry parameters after COVID-19, their exercise capacity and peak oxygen uptake remained relatively intact. Komici, Bianco [58] reported lower FEV1 in COVID-19 patients, while Nissanka, Jayasekara [59] found normal spirometry in most athletes but noted reduced mid-expiratory flow rates in some cases, indicating potential effects on peripheral airways. 

These results underscore the importance of comprehensive pulmonary assessments in athletes who recover from COVID-19 as they may reveal subtle impairments that could impact performance. Given the potential long-term implications of COVID-19 on respiratory health, ongoing monitoring and tailored rehabilitation programs are necessary to ensure optimal recovery and performance of elite athletes.

### 4.3. Autonomic Regulation

Our examination of heart rate variability (HRV) parameters did not reveal significant differences between the COVID-19 and control groups, indicating preserved autonomic function in elite athletes (Table 3). This finding is consistent with Besson, Guex [60], who reported no significant changes in HRV measures among elite athletes following COVID-19. Several factors are likely to contribute to the preservation of autonomic function.

First, training-induced cardioprotective play a crucial role. High-intensity endurance training enhances parasympathetic tone and improves the overall autonomic balance [61]. This training-induced adaptation may provide a buffer against potential COVID-19-related autonomic dysregulation. Second, the severity of COVID-19 cases in our athletic cohort was likely mild, given their young age and excellent physical condition. Mild cases may not induce significant long-term autonomic disruption, particularly in highly trained individuals [24]. Finally, athletic heart resilience plays a significant role. Cardiovascular adaptations associated with elite-level training may confer greater resilience against infectious challenges, facilitating for faster recovery of autonomic function [62]. These findings underscore the potential protective effects of high-level athletic training against COVID-19-induced autonomic dysregulation. 

Several studies have shown changes in heart rate variability (HRV) parameters among athletes with COVID-19 [12]. Sollazzo et al. [63] observed decreased parasympathetic activity and increased heart rates in elite athletes who had contracted COVID-19 compared to controls without long-term symptoms. Additionally, elite swimmers experienced reduced vagal activity during lockdowns due to decreased training volume, but their HRV returned to normal levels within four weeks of resuming training [64]. This emphasizes the adaptability of HRV in response to variations in training routines.

These findings suggest that although high-level athletic training may offer some protection against COVID-19-induced autonomic dysregulation, the multifaceted impact of the pandemic on HRV is influenced by infection severity, training disruption, and vaccination. Preserving autonomic function is crucial for athletic performance because it plays a vital role in the regulation of heart rate, blood pressure, and other physiological responses during exercise [65,66]. Ensuring that athletes maintain autonomic function is essential for their overall performance and health, emphasizing the importance of continued monitoring and appropriate training adjustments during COVID-19.

### 4.4. Arterial Stiffness and Vascular Compliance

Our analysis of the arterial stiffness and vascular compliance parameters yielded mixed results (Table 4). Arterial stiffness was assessed using various indices, with significant differences observed in the reflection index, highlighting the importance of vascular health in post-COVID athletes. Although most measures did not show significant differences between the groups, a notable exception was the reflection index, which was significantly lower in athletes with a history of COVID-19 than in their non-infected counterparts. The lower reflection index observed in the COVID-19 group suggests improved vascular compliance, which may be due to adaptive changes in the vascular system following infection. Given the high fitness level of these athletes, such adaptations could reflect a protective response, warranting further investigation. The reflection index, a measure of wave reflection from the peripheral vasculature, is influenced by the properties of both large and small arteries [67]. A lower reflection index in the COVID-19 group may suggest altered vascular function, potentially due to endothelial dysfunction or changes in microvascular structure [68]. This finding is particularly intriguing given the absence of significant differences in other arterial stiffness and vascular compliance measures, such as pulse wave velocity and augmentation index. This discrepancy implies that vascular alterations in our COVID-19 group might be subtle and primarily affect specific aspects of the vascular tree, consistent with studies showing localized vascular changes post-COVID-19 [69]. Recent studies have shown that COVID-19 can significantly affect the vascular function and arterial stiffness. COVID-19 causes early vascular aging and arterial stiffness, with vascular physiology remaining impaired for at least 12 months after infection, even in otherwise healthy adults [70]. Significant vascular alterations were observed in elite male athletes who recovered from SARS-CoV-2 infection, suggesting vascular impairment [71]. Cardiovascular magnetic resonance imaging (MRI) has been used to detect myocardial inflammation in competitive athletes and identify high-risk athletes for return to play [72]. A recent study of 30 healthy elite male athletes showed significant vascular changes after COVID-19, particularly in the increase index (Aix) and Aix normalized to a heart rate of 75 beats per minute (Aix@75). These findings indicate vascular impairment despite the absence of severe symptoms or persistent issues [73]. This is consistent with broader research indicating that COVID-19 increases arterial stiffness, as demonstrated by elevated carotid-femoral pulse wave velocity (cfPWV) in COVID-19 patients compared with controls, suggesting a potential association between the virus and increased cardiovascular risk [74]. Furthermore, a longitudinal study noted that although some vascular parameters, such as aortic pulse wave velocity (aPWV), improved over time, aPWV remained significantly higher in patients with COVID-19 even 48 weeks after infection, indicating persistent arterial stiffening [70].

The preservation of the overall vascular function is encouraging and highlights the resilience of the cardiovascular system in elite athletes. This resilience can be attributed to the protective effects of regular high-intensity training. Endurance exercise improves vascular health by improving endothelial function, reducing arterial stiffness, and promoting vascular remodeling [75]. Research indicates that athletes exhibit superior vascular function and increased arterial elasticity compared with non-athletes [76].

Our findings contribute to the growing body of literature on the vascular effects of COVID-19 on athletic populations. Some studies have reported persistent vascular dysfunction in athletes following COVID-19 infection [77], whereas others have found minimal long-term effects [78]. Rajpal and Tong [72] found evidence of myocarditis and other cardiovascular abnormalities in a subset of athletes after COVID-19. Conversely, other studies have shown a return to baseline vascular function over time [52]. The mixed results of our study aligned with this broader variability, suggesting that the impact of COVID-19 on vascular function in athletes may depend on a variety of factors, including the severity of infection, the presence of underlying conditions, and individual differences in physical conditioning and recovery.

**Figure 1:**
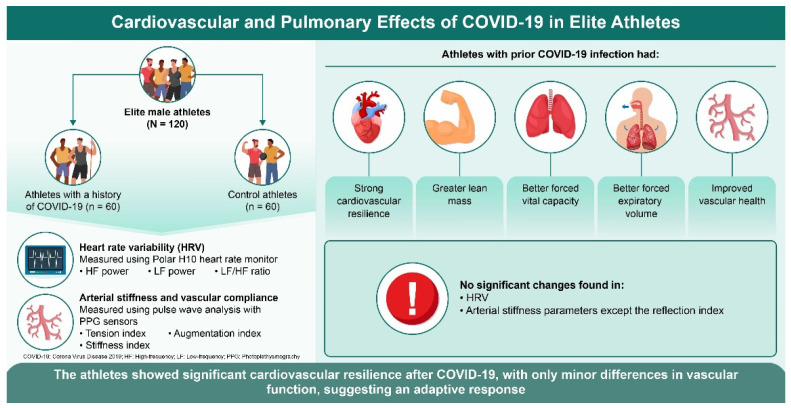
COVID-19 impacts’ graphic abstract.

In summary, although a lower reflection index in athletes with a history of COVID-19 indicates specific vascular alterations, the overall preservation of vascular function underscores the potential protective effects of regular high-intensity training. Future studies should explore these dynamics to better understand the long-term cardiovascular implications of COVID-19 on athletic populations. A graphical abstract summarizing the main results, including HRV and arterial stiffness comparisons between COVID-19 and control groups, has been added in Figure 1.

### 4.5. Hemodynamics

Our analysis revealed a significant difference in PVR between the COVID-19 and non-COVID-19 groups, with athletes who recovered from COVID-19 exhibiting a lower peripheral vascular resistance PVR (Table 5). Despite this, the two groups did not differ significantly in terms of blood pressure or cardiac output. These findings suggest that elite athletes with a COVID-19 history have adapted to maintaining cardiovascular homeostasis.

Our findings partially align with those of previous studies; however, they also present some notable differences. Some studies have reported reduced cardiac function and increased vascular impairment in non-athlete populations after COVID-19 [73,79], and our study observed preserved cardiac output in elite athletes. This finding suggests that high-performance athletic training may protect against COVID-19-related cardiovascular complications. Zacher et al. [12] also reported changes in cardiovascular autonomic function post-COVID-19, which resonates with our observation of altered PVR but not blood pressure or cardiac output.

The lower PVR observed in the COVID-19 group could indicate a compensatory mechanism for maintaining adequate tissue perfusion despite possible COVID-19-induced vascular changes [80]. Compensatory vasodilation may be an adaptive response that ensure optimal blood flow during exercise. Alternatively, combining COVID-19 exposure and continued high-level training could have prompted additional vascular adaptations, leading to a more efficient circulatory system [81]. The absence of significant differences in blood pressure and cardiac output suggests that the cardiovascular systems of elite athletes could maintain homeostasis through other compensatory mechanisms, in contrast to findings in less active populations [79]. This study indicates that rigorous training improves vascular function and elasticity, potentially mitigating COVID-19-related vascular impairment. Elite athletes showed preserved cardiac output and lower vascular resistance, suggesting cardiovascular resilience and cardioprotective benefits of sustained high-level training. These findings highlight the importance of maintaining physical activity during COVID-19 recovery and can inform rehabilitation guidelines for athletes, focusing on maintaining cardiovascular health through continued exercise.

### 4.6. Microcirculation and Oxygenation

Our analysis of microcirculatory function and oxygenation parameters revealed no significant differences between the athletes with and without COVID-19 (Table 5). Both groups exhibited similar oxygen saturation (SpO_2_) and absorption in the microcirculation system (ACI). These findings are encouraging as they suggest that elite athletes maintain robust microcirculatory function following COVID-19.

Preservation of microcirculatory health can be attributed to several factors. High-level endurance training enhances microvascular density, function, and oxygen extraction capabilities [82]. Given our athletic cohort’s excellent fitness, any acute microcirculatory disturbances caused by COVID-19 could resolve quickly, leaving no detectable long-term impact [83]. The rapid restoration of microvascular function in elite athletes may be attributable to their exceptional cardiovascular capacity and increased adaptability to physiological stressors [58,84]. The maintained microcirculatory function observed in our COVID-19 group is of significant importance, particularly considering the increasing evidence of microvascular involvement in the pathophysiology of COVID-19 [72]. Understanding these impacts is crucial for developing effective rehabilitation and monitoring strategies to ensure optimal cardiovascular health and performance. 

### 4.7. Study Limitations

A limitation of this study is that we did not consider the severity of COVID-19 or the presence of long COVID, which may influence autonomic control and HRV outcomes. Studies such as the one referenced [85] suggest that the severity of infection can have significant effects on autonomic regulation, which may explain the lack of HRV differences in our study cohort. Second, the sample size was relatively small and limited to elite male athletes, which could restrict the generalizability of the findings to other populations, including female athletes and those with varying fitness levels. Third, the study design was cross-sectional, preventing us from establishing causal relationships between COVID-19 infection and the observed cardiovascular and physiological differences. Finally, longitudinal studies tracking athletes before and after COVID-19 could provide more definitive insights into the temporal dynamics of cardiovascular changes.

### 4.8. Recommendations for Future Research

To further explore our findings, we suggest conducting long-term studies to track athletes before and after COVID-19 to understand the timeline of potential cardiovascular changes. By including thorough exercise testing, such as maximal and submaximal protocols, cardiovascular function under stress we can evaluate. This may reveal subtle variations in performance capacity and cardiovascular responses which are not obvious at rest. Furthermore, examining inflammatory markers, endothelial function indicators, and cardiac biomarkers could offer insights into observed differences and help clarify the underlying causes of COVID-19-related cardiovascular changes in athletes.

## 5. Conclusions

In summary, our study contributes to a growing body of evidence suggesting that high-level physical fitness may protect against the cardiovascular complications associated with COVID-19. Our findings highlight the remarkable cardiovascular resilience of elite athletes following COVID-19, reinforcing the importance of physical fitness in mitigating the potential long-term impacts of the virus. These insights underscore the need for ongoing monitoring and customized rehabilitation programs for athletes recovering from COVID-19 and the potential benefits of regular exercise in improving resilience against infectious diseases in a larger population.

## Figures and Tables

**Table 1:** Demographics, anthropometrics, body composition, and training characteristics of participants with and without a history of COVID-19.

	COVID-19 Group (n = 60)	Control Group (n = 60)	t	p	95% CI	Effect Size
Age (years)	23.4 ± 6.6	21.0 ± 9.1	1.65	0.101	[−0.46, 5.26]	0.30
Wt (kg)	78.4 ± 9.0	74.8 ± 12.0	1.45	0.150	[−1.31, 8.91]	0.34
Ht (cm)	183.4 ± 6.3	181.2 ± 9.0	1.55	0.124	[−0.08, 0.64]	0.63
BMI (kg/m^2^)	26.6 ± 1.1	26.6 ± 1.8	0.00	1.000	[−0.54, 0.54]	0.00
LM (kg)	68.2 ± 7.4	64.7 ± 8.5	2.41	0.017	[0.62, 6.38]	0.44
FM (kg)	12.9 ± 3.6	13.0 ± 5.8	−0.11	0.913	[−1.84, 1.64]	−0.02
TBW (L)	63.8 ± 2.7	63.7 ± 4.2	0.16	0.871	[−1.18, 1.38]	0.03
MM (kg)	23.3 ± 2.4	23.2 ± 3.4	0.19	0.849	[−0.96, 1.16]	0.03
PhA (degrees)	7.9 ± 1.2	7.7 ± 1.4	0.84	0.403	[−0.27, 0.67]	0.15
Years of training (years)	13.55 ± 4.22	12.8 ± 5.07	0.82	0.413	[−0.21, 0.51]	0.15
Hours of training per week	15.30 ± 5.19	15.89 ± 6.35	−0.56	0.578	[−0.46, 0.26]	−0.10

Notes: Values are presented as mean ± standard deviation. n = sample size; t = t-value; *p* = *p*-value; CI = confidence interval; effect size = Cohen’s d. Abbreviations: COVID-19 group (Post COVID-19 group), without COVID-19 (control group); Age: age; Wt: weight; Ht: height; BMI: body mass index; LM: lean mass; FM: fat mass; TBW: total body water; MM: muscle mass; PhA: phase angle.

**Table 2:** Pulmonary function test results in athletes with and without prior COVID-19.

	COVID-19 Group (n = 60)	Control Group (n = 60)	t-Value	*p*-Value	95% CI	Cohen’s d
FVC (L)	5.9 ± 0.8	5.3 ± 1.1	3.40	0.001	[0.24, 0.96]	0.62
FEV1 (L)	5.0 ± 0.7	4.6 ± 1.0	2.75	0.007	[0.10, 0.70]	0.49
FEV1/FVC (%)	85.0 ± 6.6	86.0 ± 11.5	−0.55	0.584	[−4.80, 2.80]	−0.10
PEF (l/s)	8.5 ± 1.5	8.8 ± 1.8	−0.90	0.370	[−1.14, 0.43]	−0.18
FEV1 %	102.9 ± 15.3	106.8 ± 13.3	−1.54	0.126	[−8.89, 1.12]	−0.27
FEF25–75% (L/s)	4.2 ± 1.0	4.5 ± 1.2	−1.38	0.171	[−0.72, 0.13]	−0.27
MVV (L/min)	180 ± 20	175 ± 25	1.23	0.220	[−3.02, 13.02]	0.22

Notes: Values are presented as mean ± standard deviation. n = sample size; t = t-value; *p* = *p*-value; CI = confidence interval; effect size = Cohen’s d. Abbreviations: COVID-19 group (Post COVID-19 group), without COVID-19 (control group); FVC: forced vital capacity; FEV1: forced expiratory volume in 1 s; FEV1/FVC: ratio of forced expiratory volume in 1 s to forced vital capacity; PEF: peak expiratory flow; FEV1 %: forced expiratory volume in 1 s as a percentage of the predicted value; FEF25–75%: forced expiratory flow at 25–75% of the pulmonary volume; MVV: maximal voluntary ventilation.

**Table 3:** Heart rate variability parameters in athletes with and without prior COVID-19.

	COVID-19 Group (n = 60)	Control Group (n = 60)	t-Value	*p*-Value	95% CI	Cohen’s d
Heart rate (bpm)	79.3 ± 9.5	81.5 ± 12.7	−1.01	0.316	[−6.51, 2.11]	−0.20
HF (%)	32.7 ± 7.1	31.4 ± 7.4	0.94	0.349	[−1.39, 3.79]	0.18
LF (%)	35.7 ± 9.1	35.1 ± 11.8	0.29	0.770	[−3.40, 4.60]	0.06
LF/HF	1.2 ± 0.5	1.2 ± 0.6	0.00	1.000	[−0.21, 0.21]	0.00
SDNN (ms)	51.9 ± 10.5	53.5 ± 16.1	−0.56	0.576	[−6.97, 3.77]	−0.11

Notes: Values are presented as mean ± standard deviation. n = sample size; t = t-value; *p* = *p*-value; CI = confidence interval; effect size = Cohen’s d. Abbreviations: COVID-19 group (Post COVID-19 group), without COVID-19 (control group); HF: high frequency; LF: low frequency; LF/HF: low-frequency to high-frequency ratio; SDNN: standard deviation of NN intervals; bpm: beats per minute; ms: milliseconds.

**Table 4:** Arterial stiffness and vascular compliance indices in athletes with and without prior COVID-19.

	COVID-19 Group (n = 60)	Control Group (n = 60)	t	* p *	95% CI	Cohen’s d
TI (unitless)	128.0 ± 56.2	128.9 ± 80.6	−0.07	0.945	[−29.66, 27.86]	−0.01
SI (unitless)	6.4 ± 0.7	6.1 ± 0.9	1.86	0.065	[−0.02, 0.62]	0.35
RI (%)	25.8 ± 2.7	27.2 ± 4.3	−2.10	0.038	[−2.73, −0.08]	−0.40
AI (unitless)	1.0 ± 0.1	1.0 ± 0.1	0.00	1.000	[−0.03, 0.03]	0.00
b/a (unitless)	−1.1 ± 0.2	−1.1 ± 0.2	0.00	1.000	[−0.08, 0.08]	0.00
c/a (unitless)	−0.1 ± 0.1	−0.1 ± 0.1	0.00	1.000	[−0.03, 0.03]	0.00
d/a (unitless)	−0.3 ± 0.0	−0.3 ± 0.0	0.00	1.000	[−0.01, 0.01]	0.00
e/a (uniform)	0.3 ± 0.1	0.3 ± 0.2	0.00	1.000	[−0.06, 0.06]	0.00

Notes: Values are presented as mean ± standard deviation. n = sample size; t = t-value; *p* = *p*-value; CI = confidence interval; effect size = Cohen’s d. Abbreviations: COVID-19 group (Post COVID-19 group), without COVID-19 (control group); TI = tension index; SI = stiffness index; RI = reflection index; AI = augmentation index; b/a, blood flow ratio; c/a, compliance ratio; d/a, dilation ratio; e/a, elasticity ratio.

**Table 5:** Hemodynamic and oxygen saturation parameters in athletes with and without prior COVID-19.

	COVID-19 Group (n = 60)	Control Group (n = 60)	t	* p *	95% CI	Cohen’s d
MBP (mmHg)	89.8 ± 8.7	89.4 ± 7.7	0.27	0.785	[−1.99, 2.59]	0.04
SBP (mmHg)	125.0 ± 12.0	124.3 ± 11.9	0.29	0.772	[−3.54, 4.94]	0.06
DBP (mmHg)	72.1 ± 8.7	71.9 ± 7.9	0.14	0.886	[−2.37, 2.77]	0.02
PVR (dyn·s/cm^2^)	1085.4 ± 123.7	1142.5 ± 166.2	−2.00	0.048	[−113.50, −0.50]	−0.37
CO (L/min)	6.7 ± 0.7	6.4 ± 0.9	1.89	0.061	[−0.01, 0.61]	0.34
SVI (mL/m^2^/beat)	3.3 ± 0.3	3.3 ± 0.4	0.00	1.000	[−0.10, 0.10]	0.00
SpO_2_ (%)	94.4 ± 1.6	94.5 ± 1.8	−0.31	0.756	[−0.85, 0.65]	−0.06
ACI (mlO_2_/min/m^2^)	71 ± 5	73 ± 7	−1.80	0.074	[−4.20, 0.2]	−0.33

Notes: Values are presented as mean ± standard deviation. n = sample size; t = t-value; *p* = *p*-value; CI = confidence interval; effect size = Cohen’s d. Abbreviations: COVID-19 group (Post COVID-19 group), without COVID-19 (control group); MBP: mean blood pressure; SBP: systolic blood pressure; DBP: diastolic blood pressure; PVR: peripheral vascular resistance; CO: cardiac output; SVI: stroke volume index; SpO_2_: blood oxygen saturation; ACI: aerobic capacity index.

## Data Availability

The datasets generated and/or analyzed during the current study are available from the corresponding author upon reasonable request.
